# Curriculum Innovation: Standardized Curriculum Improves Physician and Nurse Competence and Self-Assessment in Brain Death Determination and Counseling

**DOI:** 10.1212/NE9.0000000000200270

**Published:** 2025-11-13

**Authors:** Zachary Hodosevich, Joseph Conovaloff, Soumya Ravichandran, Jamie Nicole Labuzetta

**Affiliations:** 1 Cleveland Clinic Neurological Institute, OH;; 2 Department of Neurosciences, University of California San Diego, San Diego, CA; and; 3 School of Medicine, University of California San Diego, San Diego, CA.

## Abstract

**Background and Objectives:**

There is less familiarity with brain death/death by neurologic criteria (BD/DNC) among medical professionals when compared with cardiac death. Targeted education should be offered to providers to improve competence and familiarity with this entity. This study was designed to evaluate the impact of a standardized curriculum on physician and nurse competence and comfort regarding BD/DNC determination. Physicians will be able to define BD/DNC, understand societal perceptions of BD/DNC, evaluate the prerequisites for determination of BD/DNC, perform a BD/DNC examination, and provide bedside counseling regarding BD/DNC. Nurses will be able to define BD/DNC, understand societal perceptions of BD/DNC, evaluate the prerequisites for determination of BD/DNC, and provide bedside counseling regarding BD/DNC.

**Methods:**

BD/DNC curricula were developed and offered to (1) neurosciences and critical care physicians to enhance accurate declaration and discussion of BD/DNC and (2) critical care nurses to improve comfort and competence in answering family questions about BD/DNC at the bedside. Physicians underwent a formal didactic, followed by simulation-based training incorporating examination and communication skills. Critical care nurses underwent formalized didactic, followed by peer-based simulated conversations of frequently asked questions. All participants completed a precurriculum and postcurriculum survey and quiz. The Wilcoxon signed-rank test was used for analysis of quiz scores and Likert survey responses.

**Results:**

Forty-six neurosciences and critical care physicians (37% female) and 30 nurses (80% female) participated in their respective curriculum. Median scores on knowledge quiz significantly improved for physicians (5 [interquartile range (IQR) = 4, 6] before to 7 [IQR = 6, 8] after, *p* < 0.001) and for nurses (4.5 [IQR = 4, 5] before to 7 [IQR = 6, 8] after, *p* < 0.001). Median physician comfort in independently performing BD/DNC declaration increased (3 [IQR = 1, 5.25] before to 8 [IQR = 7, 9] after, *p* < 0.001). Median comfort in discussing BD/DNC with patients' family increased for both physicians (5 [IQR = 3.75, 7] before to 8 [IQR = 7, 9] after, *p* < 0.001) and nurses (5 [IQR = 3, 8] before to 8 [IQR = 7, 8.5] after, *p* < 0.001).

**Discussion:**

Formalized education on BD/DNC improves competence and self-assessment measures among physicians and nurses.

## Introduction and Problem Statement

Brain death, or *death by neurologic criteria* (BD/DNC), refers to the permanent comatose state after a catastrophic brain injury with complete loss of brain and brainstem function/reflexes.^1^ The Uniform Declaration of Death Act laid the legal foundation of the declaration of brain death according to accepted medical standards.^2^ Since then, several medical societies have agreed on the American Academy of Neurology (AAN)'s robust and clear guidelines for evaluation of patients with suspected brain death.^1-3^ The BD/DNC determination relies on clinical examination,^1^ and no known cases of inaccurate brain death diagnosis exist when compliant application of the guidelines is used.^4^

Despite these validated guidelines, there is variability in application due to individualized hospital policies incongruent with AAN guidelines, differing provider qualifications and training requirements (e.g., neurology vs pulmonary critical care vs neurocritical care), lack of exposure during clinical training, and public misinformation.^5-8^ This variability in BD/DNC evaluation propagates incorrect evaluation techniques and procedures.^5^ While the reasons for the variability are multifactorial in nature, there is a call for formalized training in BD/DNC evaluation to reconcile these variations and provide a higher standard of care to patients and their families.^1,9,10^

Simulation-based training efforts for brain death declaration have gained momentum in recent years to address gaps in education about BD/DNC and promote standardized training.^11^ Simulation training is uniquely positioned to provide uniform mastery of skills for high-stakes, low-frequency events. In addition to improving baseline knowledge and provider skills in BD/DNC,^12-14^ these simulation-based curricula can also be used to provide training in compassionate discussion about BD/DNC with families.^12-14^ These interventions have shown high test-retest validity among physicians of various training levels.^15,16^

Despite these educational strides for physicians, similar curricula for critical care nurses have been overlooked. Surveys of nurses in intensive care units express obscurity and concern in diagnosing, managing, and conveying the concept of brain death to patients' families,^17,18^ which can perpetuate disagreements on the treatment plan in a clinical setting^19,20^ and can allow personal beliefs and motives to seep into the diagnostic confirmation process.^21^ This lack of BD/DNC training and educational content for nurses has repercussions for clinical care, such as contributing to inadequate or inaccurate family communication.^21^

### Objectives

To address the need for formalized BD/DNC education for a multidisciplinary team, we developed a curriculum targeted toward multidisciplinary physicians (residents, fellows, and attendings) *and* critical care nurses who interface with patients undergoing BD/DNC evaluation. The curriculum used components of didactic knowledge, simulated brain death examination, and family-centric counseling on brain death. Objectives of the curriculum varied depending on the background of the participant. For physician participants, the objectives included the ability to define BD/DNC, understand the societal perceptions of BD/DNC, perform a BD/DNC examination (including evaluating the prerequisites for the examination), demonstrate familiarity with documentation of BD/DNC examination, and provide counseling to a family member regarding BD/DNC. For nursing colleagues, objectives included the ability to define BD/DNC, understand the societal perceptions of BD/DNC, understand the prerequisites for determination of BD/DNC, and provide bedside counseling regarding BD/DNC.

## Methods

### Curriculum Development

Expanding on our group's previous work,^12^ we developed and implemented brain death curricula for physicians and nurses who are most likely to interface with patients undergoing BD/DNC evaluation. The physician sessions used a 2-day, 3-hour curriculum including a 1-hour didactic session and 2-hour simulation session [with case study, a simulated BD/DNC examination, and a simulated encounter in counseling a patient's family member on brain death]. The didactic session was separated from the simulation session by at least 3 months in an effort to evaluate whether the didactic knowledge was retained despite the passage of time. This physician-oriented curriculum has been previously described.^12^

The curriculum was then adapted to a nursing audience with input from nursing leadership and stakeholders. Two different sessions were held with stakeholders and educational nursing leaders to review the physician didactic presentation and rate whether the information contained in each slide should be kept, modified, or removed; after 2 sessions, unanimity was reached. Nursing leadership was also queried about high-yield information they would further like to see included within the didactic session. Added topics were agreed on by all stakeholders. The key differences in the nurse curriculum compared with the physician curriculum related to the roles and responsibilities of each in the BD/DNC evaluation and are summarized in Table 1 alongside teaching materials (eAppendix1, eAppendix2). The nursing curriculum retained the 1-hour didactic session, although it was modified to remove details regarding apnea testing, details of ancillary testing, and documentation of brain death. Information was added regarding “What to Say” vs “What Not To Say” when talking to family at bedside about brain death. For example, this includes guidance on avoiding the terms “vegetative,” “minimally conscious,” “comatose,” etc. that can send mixed messages to patients' family. Didactic information was also added regarding ‘What To Do If You See a Brain Death Exam Being Performed Incorrectly,’ with strategies such as immediately sharing the hospital's brain death policy with the physician, or informing the second brain death examination physician. In lieu of in-depth examination simulation skills or a simulated encounter in delivering a brain death diagnosis, nursing colleagues participated in a simulated peer-to-peer discussion session focused on addressing previously identified frequently asked bedside questions regarding BD/DNC^22^ (eAppendix 3). These modifications allowed the nursing curriculum to be 2 hours in total length.

**Table 1:** Summary of Similarities and Differences Between Physician and Nurse BD/DNC Curricula

	Physicians	Nurses
Didactic session		
Definition and history of BD/DNC	Yes	Yes
Video demonstration brain death examination	Yes	Yes
Details of apnea testing	Yes	No
Ancillary testing in BD/DNC	Yes	Yes
Extracorporeal membrane oxygenation patients	Yes	No
Postarrest patients on temperature control	Yes	No
Documentation of brain death	Yes	No
Misdiagnosis and common pitfalls	Yes	Yes
Controversial cases	Yes	Yes
Family involvement	Yes	Yes
Simulation of BD/DNC declaration using manikin	Yes	No
Simulation of counseling patient's family member on BD/DNC	Yes	No
Peer-to-peer discussion of answering common questions	No	Yes

### Assessments

All participants completed a presession questionnaire before their didactic session, including previous experience with brain death, a subjective survey, and objective knowledge testing using the online platform Survey Monkey (eAppendix 4). The surveys for physicians and nurses differed slightly in content based on respective roles in patient care. The subjective survey for physicians included a 1–10 point Likert scale survey of physicians' self-assessment of their knowledge of brain death and comfort in performing brain death declaration, documenting brain death, and discussing brain death. The subjective survey for nurses included a 1–10 point Likert scale survey of nurses' feelings toward their knowledge of brain death, comfort in discussing brain death with patients' families, and comfort in discussing brain death with colleagues. This Likert scale was calibrated as 0 indicating “I know nearly nothing” and 10 indicating “I know everything there is to know.” The objective knowledge quiz included 10 questions for physicians and 8 questions for nurses. After their participation in the educational sessions, all participants completed a postsession questionnaire, which included randomized repetition of all previous questions and new open-ended questions to give feedback on the brain death curriculum. This assessment was performed immediately after the in-person session so that the impact of the teaching on knowledge acquisition could more readily be identified. The curricula and all quiz questions were developed using neurocritical care physician expert opinion; the nursing quiz questions mirrored the physician quiz questions, with input and feedback from nursing stakeholders and educators, and were systematically validated.^23,24^

### Participants

Educational sessions were offered to physicians and nursing providers/staff at an academic medical center. Physicians (residents, fellows, and attendings) within the departments of neurology, neurosurgery, and critical care who had not previously participated in our formal BD/DNC course were invited to one of several educational sessions between June 2021 and December 2022. Nurses from the neurocritical care, surgical critical care, and medical intensive care units, in addition to neurosciences float nurses, were invited to attend one of several educational sessions between August and December 2022.

### Statistical Analysis

Data were analyzed initially for normal distribution. Both Likert scale survey responses and knowledge quiz scores were treated as ordinal data and analyzed using the Wilcoxon signed-rank test. The significance level was set at *p* < 0.05. Data analysis was performed using software SPSS version 28.0.1.1 (IBM Corp. Released 2021, IBM SPSS Statistics for Windows, Version 28.0, Armonk, NY).

### Research Ethics and Informed Consent

This study (811952) was determined by the UC San Diego IRB to be exempt because of research being conducted within established educational settings and falling within normal educational practices. Informed consent to participate in this study was obtained by (1) stipulating in the recruitment messages that these sessions were part of a voluntary educational initiative and (2) participants verifying their consent (and adult age) as a clickable response preceding the Survey Monkey questionnaires used during sessions (to enter the surveys and quizzes, participants needed to click “yes” to indicate that they consented to participate).

### Data Availability

Anonymized data not published within this article will be made available by request from any qualified investigator. Any educator wishing to explore the curriculum in more detail may also contact Dr. LaBuzetta directly.

## Results and Assessment Data

In total, 46 physicians and 30 nurses participated in the curriculum as designed. Table 2 provides the demographic data for curriculum participants. Of note, only 20% of physicians had previously received informal or formal brain death training, and greater than 80% had performed 5 or fewer brain death evaluations with over one-quarter (28%) of these neurocritical care or critical care–trained physicians never having participated in any BD/DNC evaluations. The plurality of nurses had witnessed 5 or fewer BD/DNC evaluations.

**Table 2:** Demographic Data for Participants Including Physician (n = 46) and Nursing (n = 30) Colleagues

**Physician colleagues**		Nursing colleagues	
**Sex, n (%)**		**Sex, n (%)**	
**Female**	17 (37)	**Female**	24 (80)
**Male**	29 (63)	**Male**	6 (20)
**Specialty, n (%)**		**Primary unit, n (%)**	
**Neurology**	14 (30.4)	**Neuro ICU**	19 (63.3)
**Neurocritical care**	2 (4.3)	**Non-neuro ICU or wards**	11 (36.7)
**Neurosurgery**	11 (23.9)		
**Non-neurology critical care**	19 (41.3)		
**Stage in career**		**Years since completion of training**	
**Resident**	24 (52.1)	**Current nursing Student**	1 (3.3)
**Fellow**	17 (37.0)	**Less than 5 y**	4 (13.3)
**Attending**	5 (10.9)	**5–10 y**	9 (30.0)
		**More than 10 y**	16 (53.3)
**Number of previous BD/DNC declarations performed**		**Number of previous witnessed BD/DNC declarations**	
**0**	13 (28.3%)	**0**	7 (23.3)
**1 to 5**	25 (54.3%)	**1 to 5**	11 (36.7)
**6 to 10**	5 (10.9%)	**6 to 10**	5 (16.7)
**11 to 15**	1 (2.2%)	**11 to 15**	2 (6.7)
**16 or more**	2 (4.3%)	**16 or more**	4 (13.3)

Table 3 provides the median precurriculum and postcurriculum subjective Likert scores and objective knowledge quiz scores for physicians and nurses. Both groups showed statistically significant improvements in median comfort across *all* subjective domains and the objective knowledge quiz. Median scores on the knowledge-based quiz improved from 5 to 7 out of 10 for physicians and from 4.5 to 7 out of 8 for nurses (both *p* < 0.001). Median self-reported knowledge about BD/DNC improved from 4 to 8 among physicians (*p* < 0.001) and improved from 5 to 8 among nurses (*p* < 0.001). Median physician comfort in performing independent brain death declaration increased from 3 to 8, out of 10 (*p* < 0.001). Median physician comfort in documenting a brain death assessment increased from 4 to 8, out of 10 (*p* < 0.001). Median comfort in discussing brain death with patients' family increased from 5 to 8 for physicians and from 5 to 8 for nurses (both *p* < 0.001).

**Table 3:** Results From Physician and Nurse Surveys and Knowledge-Based Quizzes Before and After Participating in BD/DNC Curriculum

Variable	Median score (IQR)		Median difference (95% CI)	*p* Value
	Before curriculum	After curriculum		
Physicians				
Knowledge of BD	4 (3, 6)	8 (7, 8)	3.5 (2.5, 4.0)	<0.001
Knowledge of BD compared with colleagues	5 (4, 5)	8 (7, 9)	3.5 (3.5, 4.0)	<0.001
Comfort declaring BD independently	3 (1, 4.25)	8 (7, 9)	4 (3.5, 5.0)	<0.001
Comfort discussing BD with colleagues	6 (5, 7)	9 (8, 9)	3 (2.0, 3.5)	<0.001
Comfort discussing BD with families	5 (3.75, 7)	8 (7, 9)	3 (2.5, 3.5)	<0.001
Comfort documenting BD	4 (1, 6)	8 (7, 9)	4 (3.5, 5.0)	<0.001
Quiz score^a^	5 (4, 6)	7 (6, 8)	1.5 (1.0, 2.0)	<0.001
Nurses				
Knowledge of BD	5 (3, 6.5)	8 (7, 8.5)	3 (2.0, 4.0)	<0.001
Knowledge of BD compared with colleagues	5 (4, 7)	8 (6.5, 9)	2 (1.5, 2.5)	<0.001
Proficiency in answering family's factual questions about BD	4 (3, 7)	8 (6.5, 9)	2.5 (2.0, 3.5)	<0.001
Comfort in discussing BD with families	5 (3, 8)	8 (7, 8.5)	2.5 (1.5, 3.0)	<0.001
Comfort in discussing BD with peers	6 (3.5, 8)	8 (7, 9)	2.5 (1.5, 3.0)	<0.001
Quiz score^b^	4.5 (4, 5)	7 (6, 8)	2 (1.5, 2.5)	<0.001

Abbreviations: BD/DNC = brain death/death by neurologic criteria; CI = confidence interval; IQR = interquartile range.

a Max score 10.

b Max score 8.

Participants were asked yes/no questions regarding their own proficiency in brain death. Of the 46 physician participants who participated, only 8 (17%) reported a sense of feeling proficient in declaring brain death before participating in the course compared with self-reported proficiency in 87% (n = 40) of participants after the course. Of the 29 nurses who participated, only 13 (45%) reported feeling proficient in answering family's questions about brain death before participating in the course; after completing the course, this number increased to 25 participants (86%). Participants were asked “On a scale from 0 (not at all) to 10 (absolutely), how likely are you to recommend this course to a colleague?.” The median response among physicians was 10 [IQR = 9, 10], and the median response among nurses was 10 [IQR = 10, 10].

## Discussion and Lessons Learned

This study evaluated the impact of a formalized curriculum of BD/DNC for multidisciplinary physicians *and* critical care nurses. These learners have the highest likelihood of interfacing with BD/DNC evaluations in their practice, and this education is needed to perform their jobs at the required level from both an operational and ethical perspective. The study confirms that there are practical gaps in knowledge about BD/DNC among multidisciplinary physicians and critical care nurses, but that these gaps in performance and knowledge can be mitigated in part by a formal curriculum using multifaceted simulation training.^12,16,22^ In this study, we saw significant improvements in knowledge-based performance and self-assessment measures in both physicians and nurses after implementation of a formal brain death curriculum. These results reinforce the findings from our group's previous work with fellow-level physicians and now demonstrate the benefit of BD/DNC education for physicians at all levels of training (e.g., resident and attending), as well as for critical care nurses.^12^

Most of the included physician participants were trainees, and more than 4 of every 5 physicians had performed fewer than 5 brain death declarations. Most nurses had more than 10 years of experience; even so, 3 of every 5 nurses had witnessed fewer than 5 brain death declarations in their career. These data suggest that there is a practical unfamiliarity with BD/DNC evaluation in part secondary to limited exposure during training and the early career. Fortunately, with formalized education, our physician and nursing cohorts evidenced improved subjective and objective parameters, a finding that is consistent with previous studies of physician learners.^12,25,26^

It was felt that providing formalized education to critical care nurses was imperative. Although they do not formally conduct BD/DNC evaluations, they consistently interface with the care of the patient undergoing evaluation for BD/DNC and with patients' families. Their improved knowledge and comfort seen in this study could realistically translate into better understanding for families and real-world implications for patient care and family support. Family members spend many hours with the bedside nurse and ask them for their frank opinions and their judgments of the diagnosis, and treatment plans can change based on these interactions.^27,28^ Without education in BD/DNC evaluation, there is the risk of inaccurate communication that can contribute to mistrust and loss of the therapeutic alliance.^20,29^ Moreover, poor communication from any members of the health care team—whether from a nurse, physician trainee, or attending physician—can have practical repercussions for the medical field from a public health perspective, setting back the years of work in gaining greater societal acceptance of BD/DNC as a diagnostic entity.^30^

The process of adapting the BD/DNC curriculum from a physician audience to a nursing audience also contained valuable lessons. It was critical to include nurses in the development of this curriculum to appreciate the expected baseline level of knowledge and educational needs and to receive specific feedback on the curriculum. For instance, it would have been feasible to solely use the didactic session as the nurses' BD/DNC curriculum, or base the nurses' didactic entirely on the physician didactic session. However, there was information contained within the physician didactic that was felt to be extraneous for nursing colleagues, and removal of the simulation portion of the curriculum would have taken away nurses' opportunity to practice the material in their role-specific didactic. This was the motivation to include a peer-to-peer simulation of families' common questions about brain death. Of note, the list of common BD/DNC questions was developed from a survey where nurses were the largest respondent subgroup,^22^ which likely helped boost the realism of the session and nurse participants' satisfaction with the curriculum. Although we have included both physician (eAppendix 1) and nurse (eAppendix 2) didactic curricula within this publication, it is important to note that these materials were developed and used before the most recent release of multisociety guidelines for BD/DNC.^1^ Therefore, these materials must be carefully reviewed and updated by educators who seek to build on the curricula presented here.

One of the goals of the study was for the simulation to mirror real clinical assessment. With this aim, physician participants were allowed to use a reference for the BD/DNC criteria during the simulated examination of patients, reflecting actual clinical practice where one has access to external resources such as an institutional policy. This reference ensured that all portions of the BD/DNC examination were performed by every physician participant, but this did not negate the need for proper performance of those examination techniques. Constructive real-time feedback was given during the simulated patient experience to ensure study participants learned the proper examination techniques required in BD/DNC testing. Commentary from physician participants was that this real-time feedback feature added to the usefulness of the curriculum.

While simulation-based training in BD/DNC testing improved our learner's self-efficacy and knowledge, it is unknown for how long this improvement persists, or, indeed, what the peak subjective and objective scores might have been. However, previous literature shows that skill acquisition through simulation-based mastery learning resists decay and translates to improved patient outcomes.^31^ Future studies could assess the knowledge of BD/DNC at specific time points after formal education or in real-life observed BD/DNC evaluations, which may elucidate the opportune time for re-education. This, however, would be logistically limited by the duration of training (e.g., some critical care fellowship training programs are only 1 year) and infrequency of these BD/DNC evaluations,^32^ particularly at medical centers where a low number of BD/DNC evaluations are performed. Despite this study's unique strengths, it is important to acknowledge its limitations. First, this study was performed at a single academic health system and with health care professionals with varying levels of training. Despite this, the results were consistent with previous studies in simulation-based education in BD/DNC testing.^12,16,25^ In addition, this study used a previously published curriculum that was developed using expert input; however, the validity of the assessment tools such as Likert assessment of comfort is unknown despite their common utilization in simulation studies. Although one might argue that the curriculum used here is not generalizable, it is based on input from recognized brain death experts and follows closely with the AAN guidelines. Inherent in survey-based studies assessing familiarity and perceived comfort is the risk of response bias. Even with this potential, it is important that health care professionals feel confident in their knowledge of BD/DNC declaration and evaluation because this can affect their delivery of a diagnosis to a patient's family and alter the family's perception of their loved one's medical care.^33^ There was a concrete improvement in objective knowledge regarding brain death (even with delayed re-assessment), indicating that the improvement in comfort of the study participants was well founded. There does remain a possible confounding effect from our study participants receiving additional BD/DNC education or exposure outside of our curriculum, which could influence the postcurriculum survey and quiz results. There may also be a component of selection bias due to the curriculum being voluntary and thereby recruiting participants who are more motivated to learn about BD/DNC. Owing to the sample size of the study, it was not feasible to do subgroup analyses, although it was noted that BD/DNC education may result in differential impact among providers with varying levels of experience.

Brain death declarations need to be accurate one-hundred percent of the time. There are a limited number of opportunities for providers to participate in BD/DNC evaluation, which lends itself to having medical and nursing care teams with limited BD/DNC exposure. We show here that for multidisciplinary neurosciences and critical care physicians *and* for critical care nurses, simulation-based education of BD/DNC evaluation improves BD/DNC self-efficacy and knowledge. Ideally, a BD/DNC consortium could drive standardized education across the United States. Until then, institutions should work to provide formalized education around BD/DNC with the goal of improving their delivery of patient care and family communication.^1,10^

## Glossary

AAN: American Academy of Neurology
BD/DNC: brain death/death by neurologic criteria
IQR: interquartile range
